# A Low Power Low Phase Noise Oscillator for MICS Transceivers

**DOI:** 10.3390/s17010140

**Published:** 2017-01-12

**Authors:** Dawei Li, Dongsheng Liu, Chaojian Kang, Xuecheng Zou

**Affiliations:** School of Optical and Electronic Information, Huazhong University of Science and Technology, Wuhan 430000, China; leedavidhust@outlook.com (D.L.); m201472115@hust.edu.cn (C.K.); estxczou@hust.edu.cn (X.Z.)

**Keywords:** low power, low phase noise, oscillator, MICS

## Abstract

A low-power, low-phase-noise quadrature oscillator for Medical Implantable Communications Service (MICS) transceivers is presented. The proposed quadrature oscillator generates 349~689 MHz I/Q (In-phase and Quadrature) signals covering the MICS band. The oscillator is based on a differential pair with positive feedback. Each delay cell consists of a few transistors enabling lower voltage operation. Since the oscillator is very sensitive to disturbances in the supply voltage and ground, a self-bias circuit for isolating the voltage disturbance is proposed to achieve bias voltages which can track the disturbances from the supply and ground. The oscillation frequency, which is controlled by the bias voltages, is less sensitive to the supply and ground noise, and a low phase noise is achieved. The chip is fabricated in the UMC (United Microelectronics Corporation) 0.18 μm CMOS (Complementary Metal Oxide Semiconductor) process; the core just occupies a 28.5 × 22 μm^2^ area. The measured phase noise is −108.45 dBc/Hz at a 1 MHz offset with a center frequency of 540 MHz. The gain of the oscillator is 0.309 MHz/mV with a control voltage from 0 V to 1.1 V. The circuit can work with a supply voltage as low as 1.2 V and the power consumption is only 0.46 mW at a 1.8 V supply voltage.

## 1. Introduction

The demand for high-data-rate and low-power wireless transceivers is increasing significantly for Medical Implantable Communications Service (MICS) transceivers [1,2]. Among MICS, a small and low-power communication front-end is one of the most important parts, which is attracting more and more attention from both the academy and industry [3,4,5]. One of the key blocks in front-ends is the quadrature signal generator which is used for modulation and demodulation [6,7]. The quadrature signal generator will generate a pair of I/Q signals for phase shift key modulation in direct conversion systems. Additionally, the quadrature signals can be used to reduce the signal’s bandwidth, which can reduce the design complexity [8].

Low-power and low-phase-noise are both major concerns for the quadrature signal generator of MICS transceivers [9,10,11,12]. The power of the transmitter operating in the human body is limited to avoid heat damage to the human body. On the other hand, the accumulation of relatively high phase noise will lead to a time jitter, which will result in timing uncertainty [13,14]. Thus, it will directly affect the quality of the received or transmitted signal in a wireless communication system and lead to a damaging effect on the performance of the system.

There are several ways to generate quadrature local signals, such as using a phase shifter, an even-stage ring oscillator, a frequency divider, and a quadrature LC-VCO (composed of an inductor and a capacitor resonation tank) [6,7,8,9,10,11,12]. For high-frequency quadrature local generation, the phase shifter fails to create precise quadrature signals due to the unequal amplitude and AM-PM conversion (Amplitude modulation to Phase modulation conversion). The frequency divider uses a master-slave flipflop to divide a signal by a factor of two, and as the LO (local oscillator) oscillates at 2ω, it consumes extra power, and it may undergo a phase imbalance resulting from the deviation of the input duty cycle from 50% [6]. In recent years, LC-QVCO has attracted the interest of many researchers. It achieves a good phase noise performance by coupling two symmetric *LC*-tank VCOs to each other [7,12]. However, there are some disadvantages. For quadrature VCOs based on the LC-tank, the tuning range is relatively low (around 20%) compared to that of ring oscillators (>50%), and thereby the output frequency may fall out of the desired band. Also, for most digital CMOS processes, it is difficult to obtain a high Q (Quality) inductor for thin metal; therefore, some expensive processes may be required. Moreover, on-chip spiral inductors usually occupy a large chip area [7,12,13].

Ring oscillators can be easily integrated on-chip without any extra process requirement. They normally occupy less chip area, which improves the yield and reduces the cost. Several quadrature ring oscillators have been proposed [6,8,10,11]. However, previous works on ring oscillators were mostly focused on the power and tuning range instead of the phase noise which deteriorates the system performance.

In this paper, a fully integrated ring quadrature oscillator based on a differential pair with positive feedback is presented. A diode-connected load is used for low-voltage operation. A P-type MOS (PMOS) transistor is added to the output of the oscillator to improve the linearity of the VCO. A self-bias circuit for isolating the supply voltage disturbance is proposed, which improves the phase noise of the proposed circuit by nearly 10 dB. The chip is fabricated in the UMC 0.18 μm CMOS process, and measurement results show that it achieves low phase noise and low power consumption.

## 2. Architecture and the Key Circuits

**Figure 1:**
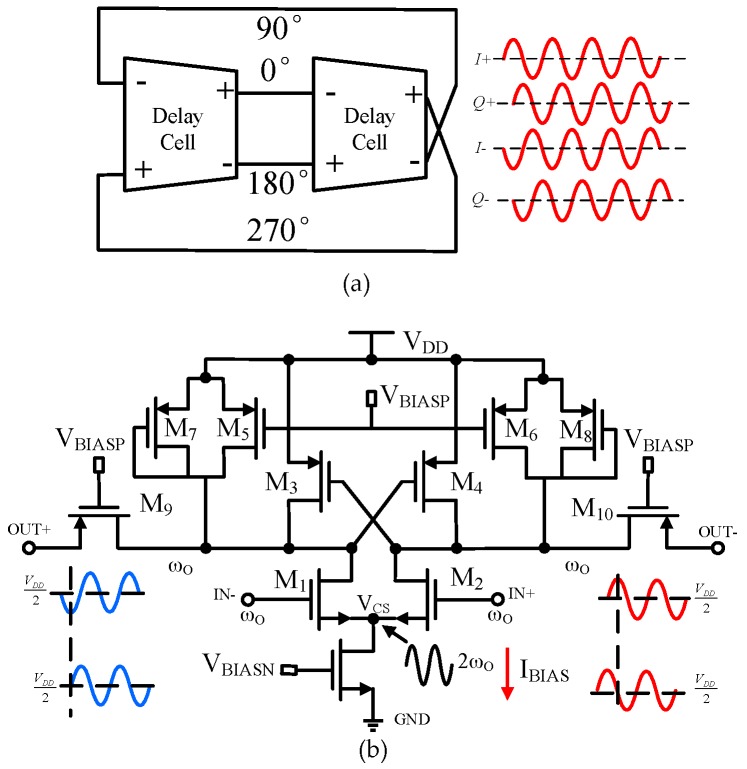
(**a**) Block-level diagram and (**b**) circuit implementation of the proposed quadrature oscillator.

The block-level diagram of the proposed quadrature oscillator and the circuit implementation of each delay cell are shown in Figure 1a,b respectively. The core of the circuits is two cross-coupled inverters. The N-type MOS (NMOS) transistors, M1 and M2, form the input pair to increase the transconductance for high-frequency operation. The two cross-coupled PMOS transistors, M3 and M4, provide negative resistance and positive feedback for the oscillation. The tuning is achieved by adjusting the gate voltage of PMOS transistors M5 and M6, while diode-connected transistors M7 and M8 serve as a load for the input. The PMOS bias voltage V_BIASP_ is nominally equal to the control voltage. The outputs of the oscillator are added to the PMOS transistors M7 and M8 to improve the linearity. The bias works in the near-threshold region to reduce the power.

**Figure 2:**
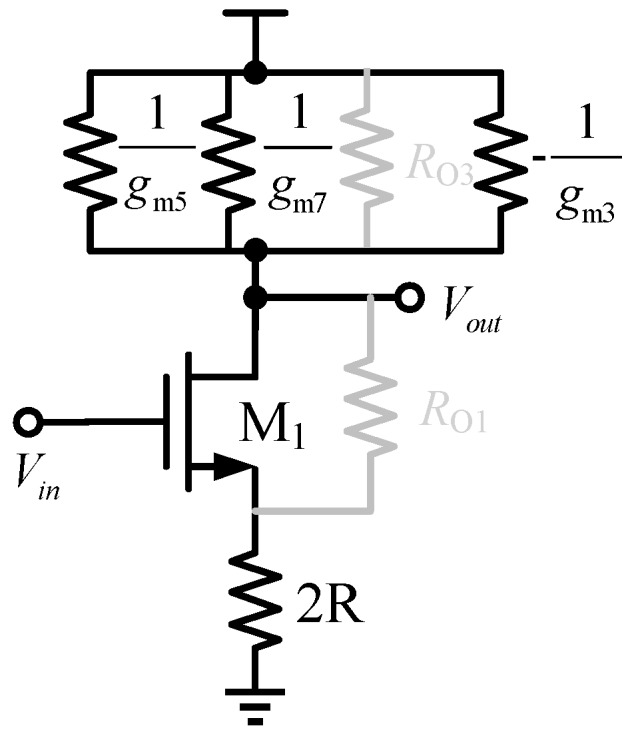
The equivalent half-circuit.

The equivalent half-circuit of the proposed oscillator is shown in Figure 2. The tail resistance is doubled according to common source point. The open loop gain of the oscillator is given by Equation (1), where *g*_m1_, *g*_m3_, *g*_m5_, *g*_m7_ are the transconductance of transistors M1, M3, M5 and M7, respectively; *r*_O3_ is the output resistance of transistor M3; R is the equivalent resistance of the bias transistor. Suppose the channel-length modulation index is negligible and *r*_O3_ approximates to infinity, then Equation (1) can be simplified to Equation (2).

(1) $$A_{V}=-\frac{g_{m1}}{1+2g_{m1}R}(\frac{1}{g_{m5}}∥\frac{1}{g_{m7}}∥r_{O3}∥\frac{-1}{g_{m3}})$$

(2) $$A_{V}=\frac{g_{m1}}{1+2g_{m1}R}\left(\frac{1}{g_{m3}g_{m5}+g_{m3}g_{m7}-g_{m5}g_{m7}}\right)$$

According to the Barkhausen criteria [15], when the total phase shift around the closed loop reaches 360° and the gain is greater than unity, the circuit oscillates. The oscillation frequency is given by Equation (3).

(3) $$F_{\mathrm{OSC}}=\frac{1}{2NR_{\mathrm{eq}}C_{L}}$$

where *R_eq_* is the equivalent output resistance and *C_L_* is the equivalent output capacitance of the delay cell. According to the equivalent half-circuit, *R_eq_* is approximately equal to *r*_O1_//*r*_O3_//*r*_O5_//*r*_O7_, where *r*_OI_ (I = 1, 3, 5,7) is the output resistance of each transistor. Neglecting *r*_O1_, *r*_O3_ and *r*_O7_ for simplification, *R_eq_* is approximately equal to *r*_O5_ and *r*_O5_ is given by Equation (4).

(4) $$\begin{array}{cc} r_{O} & =\frac{\partial V_{\mathrm{DS}}}{\partial I_{D}}=\frac{1}{\partial I_{D}/\partial V_{\mathrm{DS}}}\\ & ={\left[\lambda \cdot \frac{1}{2}\mu C_{\mathrm{ox}}\frac{W}{L}{\left(V_{\mathrm{CTRL}}-V_{\mathrm{DD}}-V_{T}\right)}^{2}\right]}^{-1} \end{array}$$

where *λ*, *μC*_ox_, *V*_CTRL_ and *V*_T_ are channel-length modulation index, the process parameter, the control voltage and the threshold voltage, respectively. According to Equations (3) and (4), the oscillation frequency varies by changing the value of *r*_O5_. When the control voltage increases, the drain current of transistor M5 decreases and *r*_O5_ increases, the oscillation frequency will decrease.

(5) $$\ell (f)=\frac{f_{0}^{2}}{f^{2}}\left(\begin{array}{c} \frac{\mu_{n}I_{\mathrm{DMn}1}+2\mu_{p}I_{\mathrm{DMp}3}+\mu_{p}I_{\mathrm{DMp}5}+\mu_{p}I_{\mathrm{DMp}7}}{I^{2}}\frac{K}{L}\frac{1}{f}\\ +2kT\gamma \frac{g_{m\mathrm{Mn}1}}{I^{2}}+\frac{kTR}{I^{2}} \end{array}\right)$$

**Figure 3:**
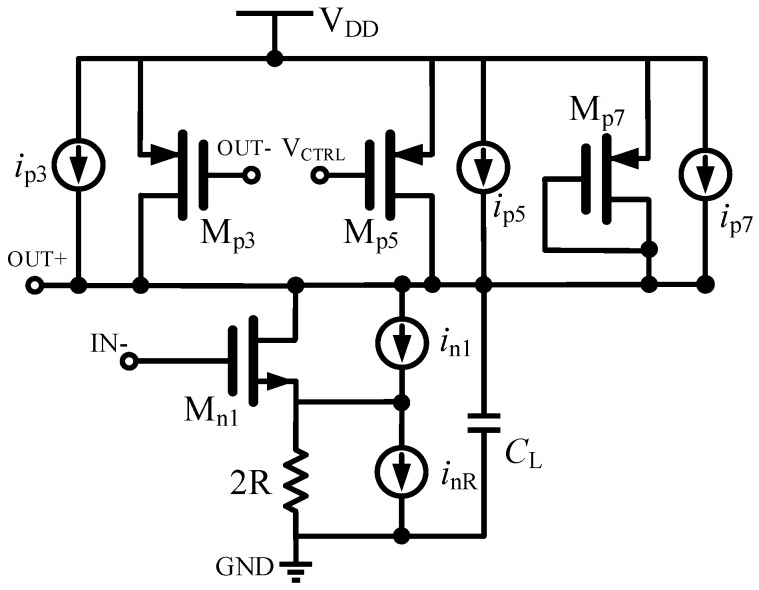
Equivalent half-circuit with noise sources.

The equivalent half-circuit with noise sources is shown in Figure 3. The relative SSB (single side band) phase noise PSD (power spectrum density) of the proposed quadrature VCO deduced from [13] is shown in Equation (5), where *f* is the offset frequency, *f*_0_ is the oscillating frequency, *I*_DM_ is the current flowing through the transistor, *I* is the current of the output node, *L* is the assumed equal channel length, *k* is the Boltzmann constant, and other coefficients *μ*, *K*, *T*, *γ* are the process relative parameters. From Equation (5), the phase noise of the proposed quadrature VCO is independent of the number of delay stages, and it depends on the oscillation frequency, the charge/discharge current of the output nodes, and the transconducdance of transistor Mn1. In Equation (5), these noise sources come from the disturbance from *V*_DD_ and the ground. If the circuit is immune to the above disturbance, the noise performance of the oscillator will be improved. Here a self-bias circuit is proposed for isolating the change in the supply and ground.

**Figure 4:**
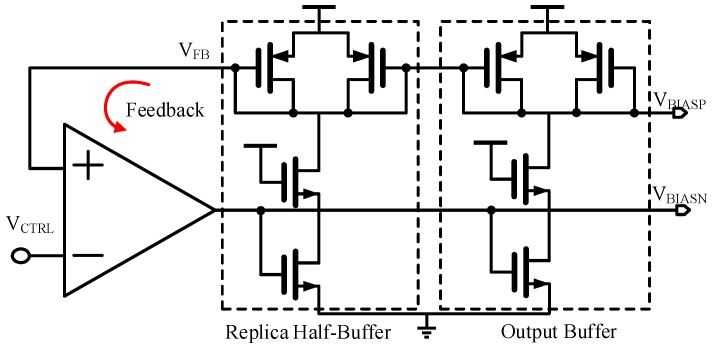
The architecture of the self-bias circuit.

The self-biasing avoids the fixed bandgap bias circuits by generating all of the internal bias voltages and currents from each other so that the bias levels are completely determined by the operating conditions. This self-biasing can also remove the constraint of the process and environment variability. By referencing all bias voltages and currents to other generated bias voltages and currents, the operating bias levels of the core are essentially established. The architecture of the self-bias circuit, shown in Figure 4, produces the bias voltage *V*_BIASP_ and *V*_BIASN_ from *V*_CTRL_. The self-bias circuit is realized with a differential amplifier and feedback buffer stages. This self-bias circuit is used to generate I_BIAS_, and the replica half-buffer stage translates this current to *V*_CTRL_ through the diode-connected device. The feedback amplifier adjusts the bias current *I*_BIAS_, and thus the voltage swing of the buffer is equal to *V*_DD_ − *V*_CTRL_. Therefore, this biasing technique dynamically adjusts the bias current in each buffer stage and hence maintains the relation *V*_DD_ − *V*_CTRL_ = *I*_BIAS_ × *R*_LOAD_ against the process and supply voltage.

**Figure 5:**
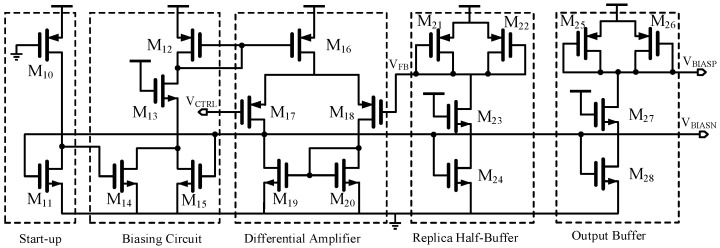
The whole self-bias circuit.

The whole self-bias circuit is shown in Figure 5. A start-up circuit composed of a PMOS transistor and a NMOS transistor drives the circuit off the degenerate point. For the rest part of the bias generator, it uses a differential amplifier to force the *V*_FB_ of the replica half-buffer equal to *V*_CTRL_, which produces the bias voltage *V*_BIASN_ of the NMOS current source, and provided the limit swing of *V*_CTRL_. A replica buffer stage is used to prevent the *V*_BIASP_ from being disturbed by the control voltage *V*_CTRL_ due to the coupling capacitance.

The biasing point of the amplifier is also produced from *V*_BIASN_, which means a self-biasing PMOS current mirror is used which utilizes the output node to produce the biasing current of the amplifier. Since the bias voltage of the core is not directly related to the supply, it is less disturbed by the noisy supply voltage, and drain voltage variations are also compensated by *V*_BIASN_.

**Figure 6:**
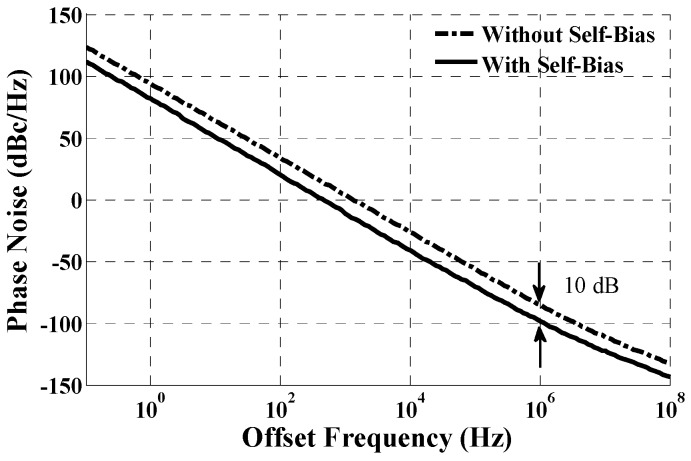
The simulated phase noise of both circuits.

The differential amplifier is realized with negative feedback architecture, and thus the frequency response and the stability should be considered. The bandwidth of the differential amplifier is set as wide as the operating frequency of the VCO so the bias can track the disturbances from the supply and ground immediately. Therefore, the main noise source of the oscillator is eliminated and the noise performance is improved. The differential structure also helps in rejecting the common noise from the substrate and supply [14]. Both optimum and non-optimum designs are simulated with Cadence Spectre for comparison. The simulation result is illustrated in Figure 6. It can be observed that the phase noise of a normal-ring VCO without self-bias is −90 dBc/Hz at a 1 MHz offset, and the phase noise with a self-bias circuit has nearly a 10 dB improvement than that of the circuit without self-bias, with only a 5% power overhead.

(6) $$d\phi =\frac{Q}{m^{2}}\cdot \frac{d\omega }{\omega_{\mathrm{OSC}}}$$

The phase mismatch of in-phase and quadrature outputs can be also analyzed. The angle variation of the quadrature output can be expressed as Equation (6), where *Q* is the quality factor of the oscillator, *ω*_OSC_ is the resonant frequency, *m* is the coupling strength between the two delay cells and *dω* is the mismatch between the resonant frequencies of the two delay cells. Equation (6) shows that dϕ is inversely proportional to the square of the coupling coefficient *m*. In this design, the negative resistance pair provides a positive feedback for the loop, and the strong rail-to-rail signal is directly injected into the input of the other oscillator. Thus, m is raised, and the phase error decreases.

## 3. Measurement Results

**Figure 7:**
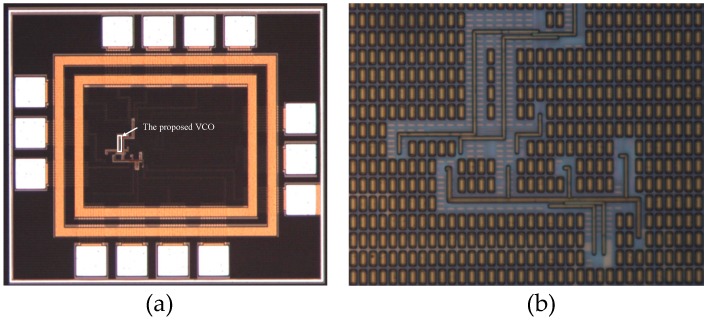
(**a**) Die photo of the whole chip; (**b**) Die photo of proposed VCO.

**Figure 8:**
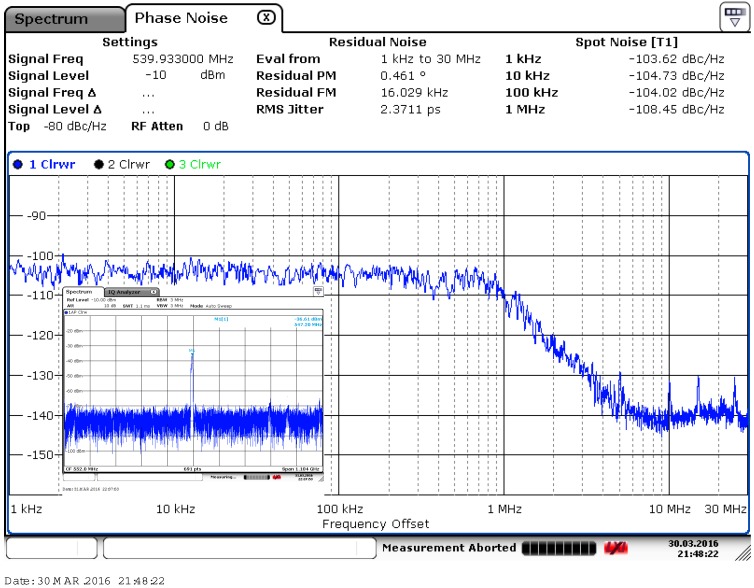
Measured phase noise of proposed VCO at 540 MHz carrier.

**Figure 9:**
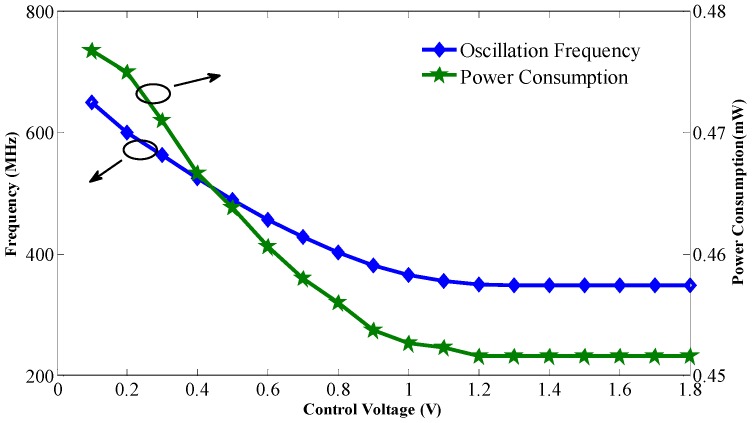
Measured tuning range and power consumption against control voltage.

**Figure 10:**
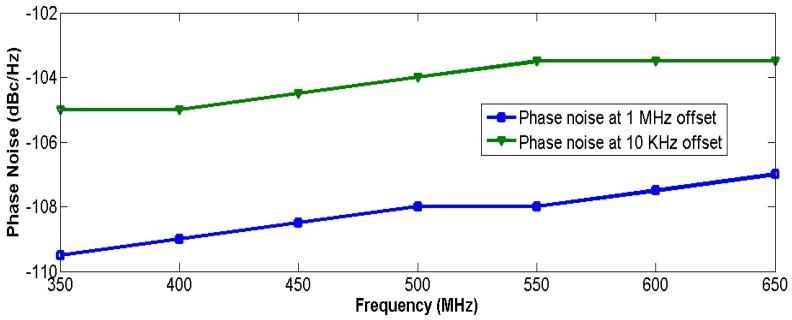
Measured phase noise at different offset frequencies.

As the circuit is sensitive to disturbances from the ground, a careful layout and shield from the ground are needed [15]. The chip is fabricated in the UMC 0.18 μm CMOS process, and the die photo is shown in Figure 7, where the core including the buffer and test circuits just occupies a 0.0006 mm^2^ area. The phase noise measured by the Rohde & Schwarz FSV7 Signal Analyzer (Rohde & Schwarz, Munich, Germany) is shown in Figure 8; it achieves −108.45 dBc/Hz at a 1 MHz offset with a center frequency of 540 MHz. The use of the self-bias circuit alleviates much of the phase noise contributed by the supply. As the supply or substrate noise is the dominant noise source of this system, the proposed VCO achieves a low phase noise that can be compared to that of LC-VCOs with a high Q resonator. The measured tuning range and power consumption are shown in Figure 9. The oscillator has a tuning range of 340 MHz from 349 to 689 MHz when the tuning voltage varies from 0 V to 1.1 V. As shown in Figure 1, when the control voltage is larger than 1.1 V, the tuning PMOS transistor will be turned off, and the frequency of the oscillator will decrease to its minimum and not change any more. The gain of the oscillator is nearly 0.309 MHz/mV with a control voltage from 0 to 1.1 V. Figure 9 shows that the power consumption is linearly proportional to the oscillation frequency and the power consumption is 453 μW at a 0.9 V control voltage. The measured phase noise at different offset frequencies is given in Figure 10. The control voltage was set from 0.8 to 0.2 V for the phase noise measurement, and it achieves a better result from a 10 KHz to a 1 MHz offset, and a phase noise degradation of up to 2.5 dB was observed at the 1 MHz offset frequency across the tuning range.

The performance comparison with previously published oscillators is given in Table 1. In Table 1, the phase noise of the proposed oscillator achieves a 20 dBc/Hz improvement compared with Reference [6] and is close to that of LC-VCOs. To fairly compare the performance of the oscillators operating at different frequencies with different power dissipation, the figure-of-merit (FoM) is used [6]. The FoM of this work has a smaller value of −166 compared to other ring-based oscillators [6,8,11,16,17]. Although the inductor- or transformer-based oscillators [9,12] show a better FoM, the chip area is inevitably large and the tuning range is also limited due to the parasitic capacitors. The quadrature phase error is dependent on the mismatch of the transistor widths of the cross-coupled pair. The phase error of the oscillator is 0.37°. Furthermore, the near-threshold bias reduces the power consumption and the power consumption is less than 500 μW. Meanwhile, it occupies the smallest area compared to prior works.

## 4. Discussion

In this paper, a low-power, low-phase-noise quadrature oscillator for MICS transceivers is proposed. For the quadrature oscillator, two major concerns exist: low power and low phase noise. First, the power of the transmitter operating in the human body is limited to avoid heat damage to the human body. Secondly, the phase noise will directly affect the quality of the received or transmitted signal in a wireless communication system. Unlike LC-VCOs with a high-quality factor resonator, the proposed oscillator is based on ring topology; as supply or substrate noise is the dominant noise source of the ring oscillator, a self-bias circuit for isolating the voltage disturbance is proposed. In this way, a good phase noise is achieved. The chip is fabricated in the UMC 0.18 μm CMOS process, and it achieves −108.45 dBc/Hz at a 1 MHz offset with a center frequency of 540 MHz. The core occupies a 28.5 × 22 μm^2^ area and the total power consumption is only 0.46 mW at a 1.8 V supply voltage. The phase noise of the proposed oscillator achieves a 20 dBc/Hz improvement compared to [6] and is close to that of LC-VCOs. The experiment results show that it is suitable for low-power implantable medical sensor applications.

## 5. Conclusions

In this paper, a low-power, low-phase-noise quadrature oscillator for MICS transceivers is proposed. The proposed oscillator generates 349~689 MHz I/Q signals, covering the MICS band. The ring oscillator is based on a differential pair with positive feedback. Each delay cell has a few transistors for low-voltage operation. A self-bias circuit for isolating the voltage disturbance is proposed, and the bias voltages created by the self-bias circuit can sense the disturbances from the supply and ground. When they are used to control the oscillation frequency, a good phase noise performance is achieved. The experimental results demonstrate that the proposed circuits achieve a good performance with a considerable reduction of the die area and power consumption, leading to simpler and more efficient designs that are suitable for low-power implantable medical sensor applications.

## Figures and Tables

**Table 1:** Comparison of state-of-the-art.

Reference	TMTT′09 [6]	TCAS I′10 [7]	TCAS I′11 [8]	TCAS I′12 [9]	TCAS II′13 [10]	TCAS II′14 [11]	TMTT′15 [12]	ISSCC′16 [16]	TCAS II′16 [17]	This Work
Supply (V)	1.3	0.6	1.8	1.2	1	0.4	0.6	0.7	0.65	1.8
Freq. (MHz)	5650	2500	1860	3600	645	350	3800	1700	400	540
Power (mW)	5	10.8	13	14–24	10	0.109	5.8–9.4	0.65	0.14	0.45–0.48
Tuning range	139.4%	9.5%	8%	46%	70%	118%	78%	68.5%	NA	65.5%
PN at 1 MHz	−88.4	−104.7	−102	−125.2	−110.8	−90	−123.7	−100.4	−90.3	−108.45
FoM	−156.5	−172	−156	−177~−185	−157	−150.5	−184	−166.9	−150.87	−166
Phase error (°)	NA	2.21	NA	<1.6	NA	NA	<1.5	NA	NA	0.37
Core Area (mm^2^)	NA	1.68 *	0.0023	0.84 *	0.02254	0.0081	0.35 *	0.003	0.0075	0.0006
Topology	Ring	PC ^a^	Ring	TB ^b^	Ring	Ring	LC-Ring	TI-Ring ^c^	F-Ring ^d^	Ring
CMOS Process	130 nm	180 nm	180 nm	130 nm	65 nm	65 nm	65 nm	65 nm	180 nm	180 nm

* Area with pads ^a^ LC-VCO Passive coupled; ^b^ Transformer-based LC-VCO; ^c^ Time-interleaved ring; ^d^ Feedforward ring VCO. $FoM=PN-20\cdot \log\left(\frac{F_{\mathrm{osc}}}{F_{\mathrm{offset}}}\right)+10\cdot \log\left(\frac{P_{\mathrm{diss}}}{1\mathrm{mW}}\right)$.
